# Digestion dynamics in broilers fed rapeseed meal

**DOI:** 10.1038/s41598-019-38725-1

**Published:** 2019-02-28

**Authors:** E. Recoules, M. Lessire, V. Labas, M. J. Duclos, L. Combes-Soia, L. Lardic, C. Peyronnet, A. Quinsac, A. Narcy, S. Réhault-Godbert

**Affiliations:** 1 BOA, INRA, Université de Tours, 37380 Nouzilly, France; 2 PRC, INRA, CNRS, IFCE, Université de Tours, 37380 Nouzilly, France; 3 Plate-forme de Chirurgie et d’Imagerie pour la Recherche et l’Enseignement (CIRE), Pôle d’Analyse et d’Imagerie des Biomolécules, INRA, Université de Tours, CHRU de Tours, 37380 Nouzilly, France; 4 Terres Univia, 11 rue de Monceau, 75008 Paris, France; 5 Terres Inovia, 11 rue Monge, Parc industriel, 33600 Pessac, France

## Abstract

Rapeseed proteins are described to be poorly digestible in chickens. To further identify some molecular locks that may limit their use in poultry nutrition, we conducted a proteomic study on the various chicken digestive contents and proposed an integrative view of the proteins recruited in the crop, proventriculus/gizzard, duodenum, jejunum, and ileum for digestion of rapeseed by-products. Twenty-seven distinct rapeseed proteins were identified in the hydrosoluble fraction of the feed prior ingestion. The number of rapeseed proteins identified in digestive contents decreases throughout the digestion process while some are progressively solubilized in the most distal digestive segment, likely due to a combined effect of pH and activity of specific hydrolytic enzymes. Fifteen chicken proteins were identified in the hydrosoluble proventriculus/gizzard content, including chymotrypsin-like elastase and pepsin. Interestingly, on the 69 distinct proteins identified in duodenum, only 9 were proteolytic enzymes, whereas the others were associated with homeostasis, and carbohydrate, lipid, vitamin and hormone metabolisms. In contrast, chicken proteins identified in jejunal and ileal contents were mostly proteases and peptidases. The present work highlights the relevance of using integrative proteomics applied to the entire digestive tract to better appreciate the protein profile and functions of each digestive segment.

## Introduction

Poultry nutrition relies essentially on the use of tremendous amounts of imported soybean by-products while its production in non-European countries and transportation to Europe are deeply associated with negative environmental impacts^1^. In the meanwhile, oleaginous crops including rapeseed are extensively cultivated in Europe. Rapeseed is essentially used for oil production and more recently, it gained interest for biofuel production, whose process generates large amounts of a co-product, rapeseed meal (RSM) that is currently used for animal feed. In contrast to other more local plants of which production remains marginal, RSM incorporation in poultry feedstuff would be very promising because of a high availability for the feed manufacturer (2 million tons a year produced in France) and high protein content (34%). However, RSM contains anti-nutritional factors (glucosinolates) that still limit the potential of this protein source in the chicken diet^2^ and rapeseed proteins incorporated in broiler diets remains poorly digestible as compared with soybean proteins^3,4^. This difference in nutritional values of RSM-based diets can be partly explained by some differences in its chemical composition compared to soybean meal (SBM) but also by the presence of major proteins including cruciferin proteins that may resist proteolysis by physiological digestive enzymes^5^. In parallel, a low digestibility of the protein source is also associated with higher amounts of undigested proteins released in the environment. Such characteristics are thus associated with major economic losses together with an overall negative environmental impact. It is well known that napin proteins contained in rapeseed are only partly digested, as these proteins (entire or partly digested) have been recovered in the ileum, the most distal segment of the digestive tract. Their presence at the end of the digestive process implies that the amino-acids contained in these protein products are not accessible to digestive enzymes and consequently are lost for animals. Except cruciferin-derived proteins, there are only few data related to the other rapeseed proteins that potentially restrain digestion. With the advent of the high throughput genome annotation combined to in-depth bioinformatic analyses, a total of 12208 distinct proteins have been identified so far in *Brassica napus* genome (05-02-2018). The progressive increase in protein accession numbers in databanks will probably allow for the identification of other anti-nutritional factors that are still uncharacterized and that may also alter digestive functions. The interaction of these plant proteins with the enzymes secreted by the digestive tract within each segment and not only the ileum, is also very important to better appreciate the dynamics of protein digestion. The catalog of proteins/enzymes that participate in chicken digestive processes is not yet complete, although some recent proteomic approaches on jejunum^6^ and ileum^7^ allowed the identification of many other proteins in addition to the well-known pepsin, chymotrypsin, trypsinogens and amylase^8–13^. The activity and function of most of these emerging molecules are still predicted based on homologies with bovine and porcine species, and their secretion by each digestive segment in chicken species have not yet been investigated.

In this article, we explored the kinetics of digestion in the crop, the proventriculus/gizzard, duodenum, jejunum and the ileum, up to three hours after feed withdrawal. The protein composition of the various digestive contents was analyzed by proteomics and the activity of proteolytic enzymes was assessed by zymography at physiological pH. Finally, an integrative comparison between all compartments was performed to better appreciate their respective specificity and function. Altogether, these results provide interesting data about some molecular physiological specificities associated with each digestive segment but also shed light on some critical factors resulting in an incomplete digestion of rapeseed-based meals, which may eventually alter the full functionality of the digestive tract. Such approach can be very promising to characterize the digestive efficiency of some new/alternative protein sources including more unusual plants, seaweeds but also insects, and may help to better evaluate their potential and their relevance in poultry nutrition, while maintaining the physiological and molecular integrity of the digestive tract. A better characterization of the interaction between the feed and the proteins recruited for digestion is indeed required to have a full representation of benefits/risks of such new nutrition strategies.

## Results

### Feed intake during synchronized feeding, ileal nitrogen digestibility, and pH of digesta

**Figure 1:**
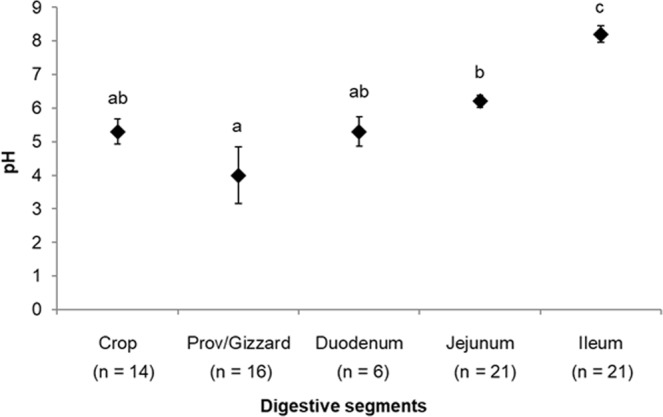
pH of the digesta within each digestive segment (Crop, Proventriculus/gizzard, Duodenum, Jejunum and Ileum). Means followed by distinct lowercase letters differ significantly (P < 0.05, refer to Methods).

The mean feed intakes over the 3 hours of synchronized feeding before euthanasia were 35 ± 6.7 g; 31 ± 5.7 g and 34 ± 3.6 g for groups A (euthanized at the end of feeding), B (euthanized 1h30 after feed withdrawal) and C (euthanized 3 h after feed withdrawal), respectively. Individual data (n = 8) are reported in Supplementary Figure 1a. This parameter was measured to check the homogeneity of the three groups and will not be further discussed. The apparent ileal digestibility of nitrogen (all groups included) was 72 ± 5.3%. The value of pH of each digesta varied between 4.0 ± 0.85 (Proventriculus/Gizzard) for the lowest value to 8.2 ± 0.25 (Ileum) for the highest (Fig. 1). Animals’ performance is reported in Supplementary Figure 1b.

### Protein concentration in the supernatant and insoluble fractions of digestive contents

**Figure 2:**
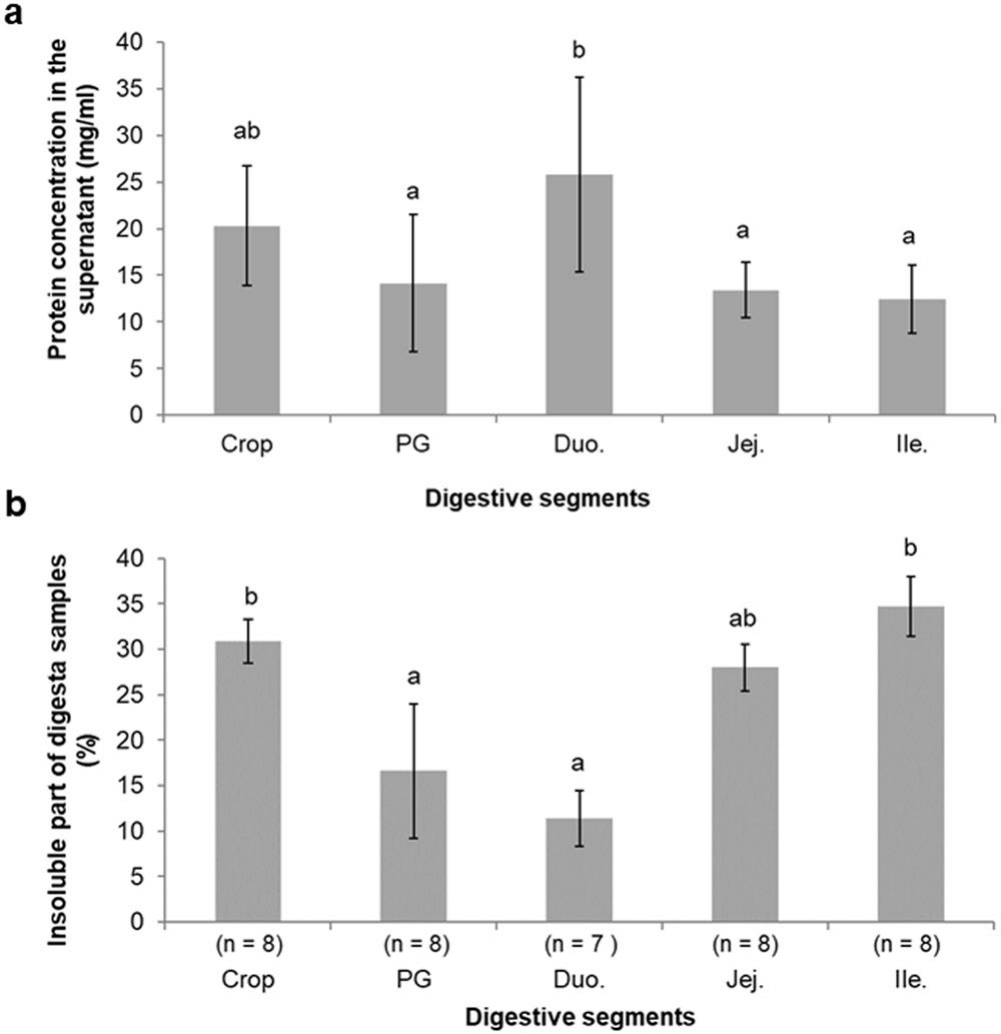
Protein concentration of hydrosoluble fractions in each digestive segment (**a**) and relative content of the insoluble fraction (**b**). Protein concentration (mg/mL) of each insoluble fraction (group A only) was determined using Dc-Biorad Assay (Bio-Rad, Marnes-la-Coquette, France) as described in Methods. The remaining insoluble fraction obtained after centrifugation was expressed as a percentage of the initial weight (group A only).

Due to a lack of digesta for some crops in groups B and C, comparison of protein concentration between each digestive segment was performed for group A only. Results are shown in Fig. 2. Protein concentration in the supernatant ranged from 12.4 ± 3.7 mg/ml (Ileum) to 25.8 ± 10.5 mg/ml (Duodenum). The protein concentration in the duodenum was significantly different from those of the proventriculus/gizzard, jejunum and ileum samples (Fig. 2a). The relative amount of the insoluble part of samples for the group A ranged between 11.4 ± 3.1% (Duodenum) to 34.7 ± 3.3% (Ileum). It decreased from crop to duodenum and increased in jejunum and ileum (Fig. 2b). Overall, these results demonstrate that digestive contents mainly consist of soluble components (which is particularly obvious in the duodenum segment). The high amount of insoluble components at the end of the digestion process (undigested components), is due to the progressive absorption/assimilation of soluble molecules by the digestive mucosa. As expected, when comparing the three A, B, C groups, protein concentration is lower 3 hours after feed withdrawal, likely resulting from protein hydrolysis followed by a progressive assimilation/absorption of protein-derived peptides and amino acids (Supplementary Figure 2a). In contrast, the insoluble part remains roughly similar, regardless of the digestive segment. These data suggest that there is a significant fraction of the feed, which is not absorbed until the end of the digestive process (Supplementary Figure 2b).

### SDS-PAGE and zymography analyses of digestive contents

**Figure 3:**
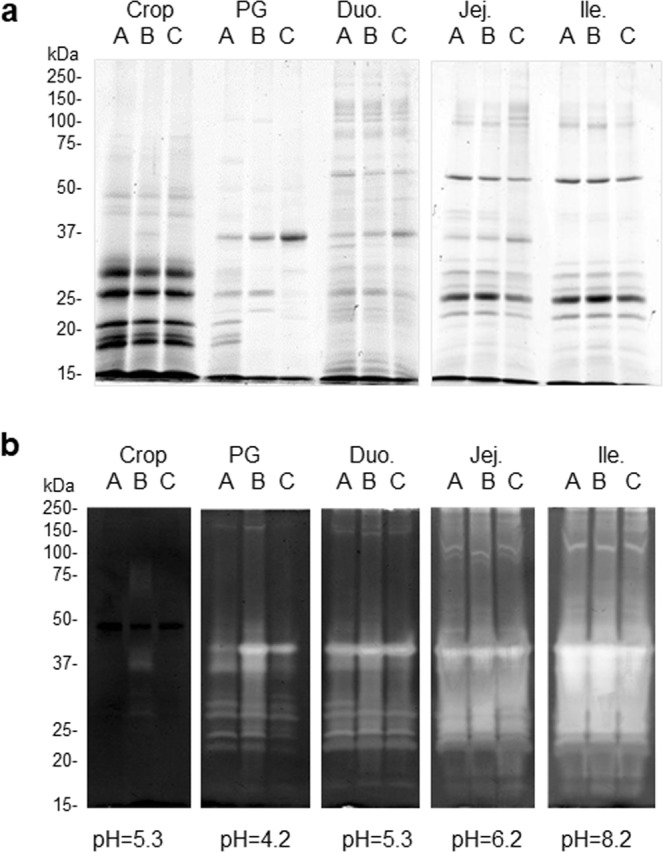
SDS-PAGE analyses (**a**) and gelatin zymographies (**b**) of digestive contents (hydrosoluble fraction) along the chicken digestive tract. SDS-PAGE were performed under reducing/denaturing conditions (40 µg proteins). Zymographies were conducted under non-reducing/non-boiling conditions (5 µg of proteins) at physiological pH (pH = 5.3 for crop; 4.0 for prov./giz; 5.3 for duo., 6.2 for jejunum and 8.2 for ileum). A, B, C correspond to group A (t = 0 after the end of feeding), B (t = 1h30 after the end of feeding) and C (t = 3 h after the end of feeding), respectively. Prov./giz, proventriculus/gizzard; Duo., duodenum; Jej. Jejunum. Crop/PG/Duo samples and Jej/Ile samples were run on two separate gels, and one empty lane was included between two sets of A, B, C groups to avoid any contamination from one set of samples to another.

Electrophoretic analyses performed on each digestive tract segment and A, B, C groups are reported in Fig. 3a. Seven bands of high to moderate intensity (17, 18, 22, 26, 30 and 48 kDa) were visible in the crop of birds. Moreover, no significant difference in relative abundance and profile was detectable between each A, B, and C group, suggesting that dietary proteins are not hydrolyzed in the crop and that, even 3 hours after the last meal since the crop of some animals still contains rapeseed feedstuff. However, it is notable that in the crop, only few samples could be collected for groups B (n = 4) and C (n = 2) as most animals from these two groups exhibited empty crops. The protein profile of this segment is quite different as compared with the others (proventriculus/gizzard, duodenum, jejunum, and ileum). In the proventriculus/gizzard digesta of group A (t = 0, corresponding to the end of the 3 h feeding period immediately followed by euthanasia), bands that were detectable in the crop profiles were still visible although they progressively disappear as the digestion continues (groups B and C). A band at 36 kDa appeared progressively and seems to exhibit a stronger signal in group C. In the duodenum, many bands of moderate intensity were visible at various molecular weights ranging from 10–15 kDa to 250 kDa and no significant difference could be detected in protein profiles between A, B, C groups. In the jejunum and ileum, profiles were very comparable with main bands detectable at about 24, 26, 30, (36 jejunum only) and 55 kDa, regardless of the group.

Proteolytic activities in all digestive contents in each A, B, C group was assessed in parallel using gelatin zymography (Fig. 3b). All zymographies were performed using 5 µg of proteins and at the physiological pH to better appreciate which proteases exhibit significant activity at a pH, which is consistent with the intrinsic physiological pH of each distinct digestive segment. These physiological pH values were obtained based on the mean pH values that were reported in Fig. 1. In the crop, a faint proteolytic band is detectable around 37 kDa for group B only. Many hydrolytic bands were noticeable in the proventriculus/gizzard with higher intensities observed in group B. It seems that the proteolytic intensity progressively increases from duodenum to jejunum and ileum.

### Mass spectrometry analyses of digestive contents

For protein identifications, protein samples of each digestive content (3 groups × 5 digestive segments + the feed itself) were analyzed by mass spectrometry using GeLC-MS/MS strategy (each sample was included in polyacrylamide gel by SDS-PAGE without fractionation, stained by Coomassie blue and in-gel digested by trypsin before nanoLC-MS/MS analyses) as described in Methods. The results were analyzed using NCBIprot_Brassica, NCBIprot_Chordata and NCBIprot_Bacteria databases.

**Figure 4:**
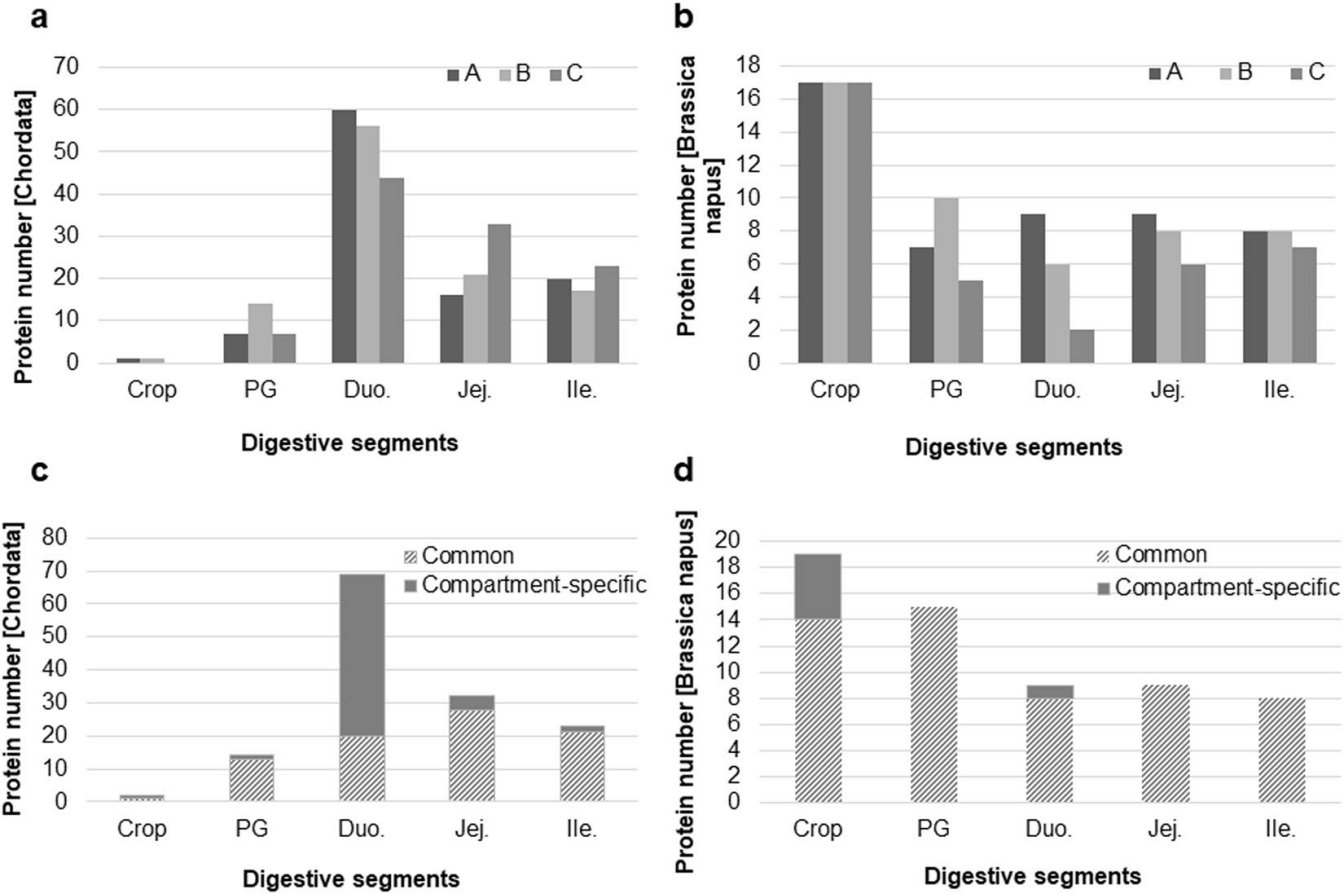
Comparative analyses of proteins identified in each digestive segment by proteomics. The total number of *Gallus gallus* proteins and *Brassica napus* proteins identified in each digestive content is presented in (**a,b**) respectively. Proteins (**c**, *Gallus gallus*, and **d**, *Brassica napus*), which were identified in at least two compartments (all A, B, C groups included) and those that were compartment-specific are shown in hatched and grey, respectively.

Eighty-six distinct *Gallus gallus* proteins were identified when combining all results from all segments (supplementary dataset 1-Chordata, sheet 1). Only two chicken proteins were identified in the crop (HBBA, GeneID 396485 and pepsin A precursor, GeneID 395691). However, the significance of these proteins in the crop remains controversial as they most likely reflect some collecting bias (blood contaminant for HBBA, which derives from hemoglobin, and reflux from the proventriculus/gizzard for pepsin). Therefore, these two proteins will not be further discussed in the present study. Up to 15 distinct proteins were identified in proventriculus/gizzard, with chymotrypsinogen 2-like precursor (GeneID 431235), pepsin A (GeneID 431235), trypsin II-P29 precursor (GeneID 396344), being the most abundant proteins (supplementary dataset 1-Chordata, sheet 1). In this segment, the highest number of proteins was identified in group B (1h30 after the 3-hour feeding period) (Fig. 4a). Sixty-nine distinct proteins were identified in the duodenum (supplementary dataset 1-Chordata, sheet 1), with a gradual decrease of the number of proteins as the digestion progresses (Fig. 4a). In jejunum, the number of proteins secreted by the chicken increases sensibly between groups A and C (Fig. 4a) with a total of 33 proteins identified within the time window studied. Twenty-three distinct proteins were identified in the ileum segment with a slight increase of the number in group C (Fig. 4a). A comparison between all segments using normalized emPAI index reveals that proteins present in the crop are actually recovered in the next segments and that the duodenum segment is the tissue, which displayed the highest number of segment-specific proteins (Fig. 4c; proteins that were identified in at least two digestive segments are shown in the hachured bars). Interestingly, the proteomic profiles of jejunum and ileum digesta are very similar (Supplementary dataset 1), which corroborates the very comparable SDS-PAGE and zymography profiles described above (Fig. 3a,b, respectively).

The analysis of the soluble fraction of the feed allowed the identification of 38 distinct proteins derived from *Brassica napus* databank (Table 1, Supplementary dataset 2-Brassica, sheet 1). As expected, the crop contains the highest number of proteins identified when compared with the other digestive segments, and except for the proventriculus/gizzard, the number decreases with time of digestion (Group A *versus* group C, Fig. 4b). Integrative analysis of results between segments revealed that most of the proteins that are not fully digested in the duodenum are indeed still recovered in the ileum digesta even after 3 hours of digestion (Fig. 4b,d). This observation gives evidence that at least 9 rapeseed proteins (hachured bars) resist proteolytic degradation up to three hours of digestion: Rapeseed trypsin inhibitor 3, CAJ44307.1; PREDICTED: putative phosphatidylglycerol/phosphatidylinositol transfer protein DDB_G0282179, XP_013714672.1; GDSL esterase/lipase At1g54020-like precursor, NP_001302825.1; PREDICTED: trypsin inhibitor DE-3-like, XP_013713143.1; BnaA08g13680D, CDY14237.1; Cruciferin subunit, AAK07609.1; BnaA06g36310D/cruciferin precursor, CDY22309.1; BnaA10g02240D, CDX89858.1; BnaA09g04300D, CDY19777.1). Sequence alignments of all these nine proteins reveal that they are indeed distinct proteins (not shown).

**Table 1:** *Brassica napus* proteins identified in the chicken digestive tract. PG, proventriculus-gizzard; Duo, duodenum; Jej, jejunum; Ile, ileum.

Protein name (accession number)	Functional domains	Digestive segment
Myrosinase-binding protein 2-like isoform X1 (XP_013655533.1); Myrosinase-binding protein related protein, partial (AAC08051.1); Myrosinase-binding protein (AAC08048.1);	Jacalin (pfam01419)/Lectins	All
12S seed storage protein CRD (XP_013664846.1); BnaA08g13680D (CDY14237.1); Cruciferin subunit (AAK07609.1); BnaA06g36310D/cruciferin precursor (CDY22309.); BnaA10g02240D (CDX89858.1)	PLN00212 super family (cl28274) /Glutelin; Provisional	All
BnaA09g04300D (CDY19777.1); BnaC09g03700D (CDX87055.1);	MATH (meprin and TRAF-C homology) domain (cd00121)	All
Probable mediator of RNA polymerase II transcription subunit 37c (XP_013648226.1); Heat shock 70 kDa protein 5-like (XP_013641680.1)	HSP70 super family (cl26953). Hsp70 chaperones/Protein folding	Duo
Napin-like (XP_013746924.1); Napin embryo-specific-like (XP_013681845.1); Napin large chain L2A (AAB37416.1); Napin large chain L2C (AAB37418.1)	AAI_SS (cd00261)/Alpha-Amylase Inhibitors (AAIs) and Seed Storage proteins (SS) participate in natural defenses of plants against insects	Crop, PG, Duo
BnaA08g15380D (CDY24772.1) ; BnaA07g13950D (CDY04569.1) ; BnaA01g24560D (CDY34239.1) ; Provicilin-like (XP_013641965.1)	Cupin_1 (pfam00190); cupin_like super family (cl21464). 11S and 7S plant seed storage proteins/major nitrogen source for the developing plant	Crop, PG
BnaC09g48200D (CDX99015.1)	Pepsin_retropepsin_like super family (cl11403). Pepsin-like aspartate proteases found in mammals, plants, fungi and bacteria. Proteolytic degradation	Crop, PG
Putative lactoylglutathione lyase (XP_013657455.1)	PLN02300 Neuromodulin_N super family (cl26511). Methylglyoxal detoxification	Crop
Superoxide dismutase [Mn] 2, mitochondrial (XP_013663290.1)	Sod_Fe_C super family (cl27368)/Detoxification of superoxide radicals	Crop
Uncharacterized protein LOC106386443 (XP_013681750.1)	Protein of unknown function (DUF1264) (pfam06884)	Crop
Phylloplanin-like (XP_013663791.1)	Pollen_Ole_e_I super family (cl03128)	All except Ile
BnaC01g26830D (CDY17559.1)	Classical (c) SDR, subgroup 1 (cd05355)	Crop
Acyl-binding/lipid-transfer protein isoform III (AAB33170.1)	nsLTP1 (cd01960)/Transfer of lipids and steroids and defense of plants	Crop, Jej
Rapeseed trypsin inhibitor 3 (CAJ44307.1)	Toxin_3 (pfam00537). Scorpion toxin-like domain; Include neurotoxins, plant defensins and small inhibitors	All
Putative phosphatidylglycerol/phosphatidylinositol transfer protein DDB_G0282179 (XP_013714672.1)	PG-PI_TP (cd00917). Bind phosphatidylglycerol and phosphatidylinositol.	All except crop, Duo
GDSL esterase/lipase At1g54020-like precursor (NP_001302825.1)	SGNH_hydrolase super family (cl01053)/Carbohydrate and lipid metabolisms	All
Trypsin inhibitor DE-3-like (XP_013713143.1)	Kunitz legume (pfam00197) Protease inhibitor/Regulation of proteolysis	All

It is notable that in our conditions, no related bacterial proteins were retrieved by mass spectrometry analysis when the database interrogation was made against NCBIprot_ Bacteria.

## Discussion

Efficiency of digestion is an intimate interplay between genetics, nature of diets and physiological status of the animal (age, health, etc.)^14–17^. The composition of the diet and the protein source have long been shown to impact performance of animals, nutrient utilization, development and physiology of the gut and microbiota profile of the intestine. Soybean meal is currently the most efficient plant-based protein incorporated in the diet of chickens but there is an increasing interest in the development of alternative feedstuffs. Among these, rapeseed has been recognized as a highly concentrated protein source with a well-balanced amino acid profile. However, the use of new protein sources is sometimes limited due to the presence of anti-nutritional factors, whose activity depends both on the nature and the processing of the feed and the physiology of the digestive tract^2,18–20^. The function of the digestive tract is to ingest, moisturize, acidify, grind the feed into small particles, and finally cleave nutrients into some small molecules that can be further assimilated by digestive mucosa. In this respect, each digestive segment has its specific function. In chickens, the digestive tract can be divided into six distinct physiological and functional segments: the crop, the proventriculus/gizzard, the duodenum, the jejunum, the ileum and the caecum^10,21^. In the present study, we explored the fate of rapeseed proteins during digestion, starting with the ingestion to the most distal part of the intestine, and the molecular response of the chicken digestive tract to digest such plant proteins. The content of the various digestive segments of animals fed rapeseed-based diet was collected at the end of a 3 hour-feeding (Group A), 1h30 (Group B) or 3 hours after feed withdrawal (Group C), and was submitted to various biochemical analyses (SDS-PAGE, proteomics, zymography). To avoid any bias of our data interpretation and to our experimental procedure, we first measured various parameters such as feed intake, pH of digesta (Fig. 2), apparent ileal digestibility of nitrogen (Supplementary Figure 1), which all appeared to be consistent with literature^22–25^ and that exhibited no statistical difference between A, B, C groups.

The main role of the crop is to store and moisturize the feed after ingestion prior to its transit towards the proventriculus-gizzard segment, without any significant secretion of endogenous proteins reported. SDS-PAGE analysis of the crop content revealed the presence of many proteins showing an apparent molecular weight <37 kDa (Fig. 3a). These proteins were essentially rapeseed-derived proteins (Fig. 4a versus b). Additionally, we could not detect any proteolytic activity using zymography approach (Fig. 3b). These data support that this segment does not likely secrete proteases nor other proteins. It is noticeable that in our experiment most animals exhibited empty crops three hours after feed withdrawal.

**Figure 5:**
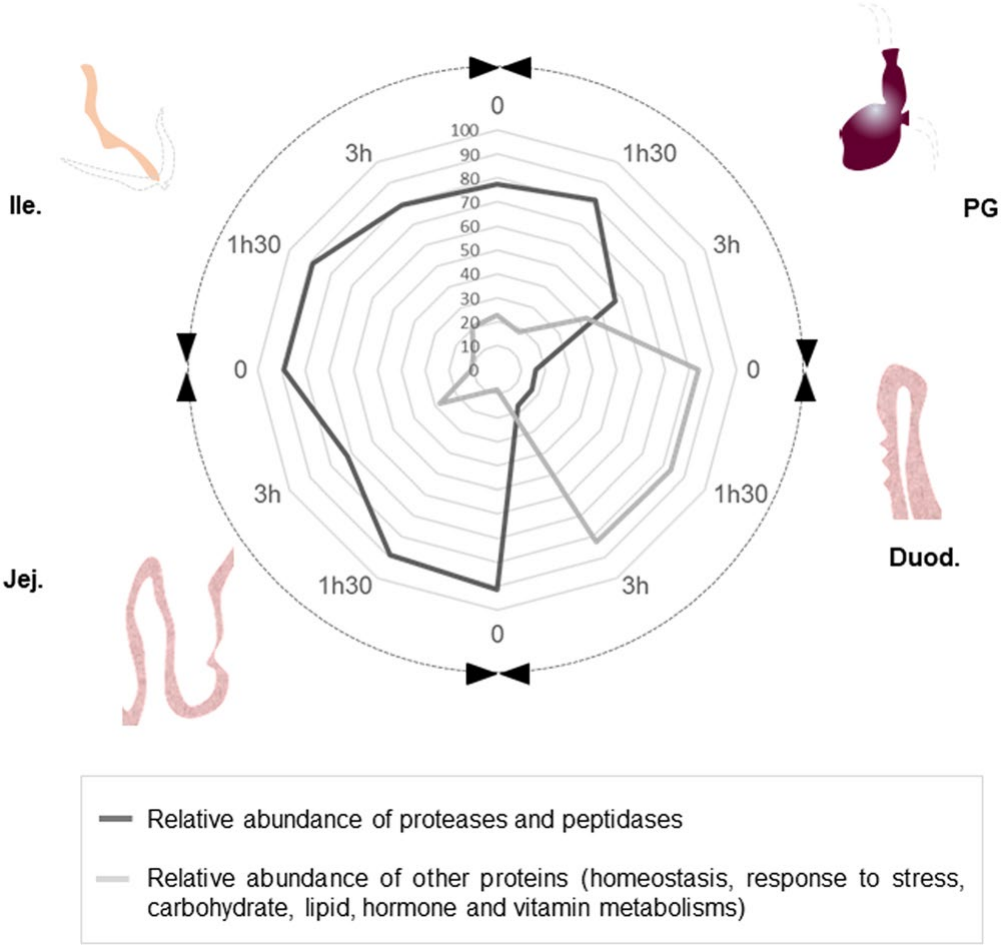
Radar diagram illustrating the kinetics of appearance/disappearance of proteases and other proteins along the digestive tract. This representation is extracted from supplementary dataset 1.

In birds, the proventriculus/gizzard refers to mammalian stomach. As a muscle, it participates in grinding and mixing ingested feed to facilitate the activity of endogenous enzymes^26^. It contains an acidic secretions (pH around 4, Fig. 1) as previously reported^26^ and essentially secretes proteases (Fig. 5) that were shown to be active (Fig. 3b). Indeed, on the total number of proteins identified in this segment, the majority were proteases (A: 4/7; B: 9/14 and C: 3/6, Fig. 5). The presence of many proteolytic enzymes highlights the importance of this segment in protein digestion. Main proteases were Pepsin A precursor (Gene ID395691), Trypsin II-P29 precursor (GeneID 396344) and Chymotrypsinogen 2-like precursor (Gene ID 431235). Pepsin is released by the chief cells in the proventriculus, auto-activates in an acidic environment (lowest pH of the digestive tract), and degrades dietary proteins into peptides^9^. Trypsin and chymotrypsin are described as pancreatic proteases that are likely recovered in the proventriculus/gizzard due to refluxes from duodenum, which are part of the normal digestive process^27^. On the 14 distinct proteins identified in the proventricular/gizzard, two were specific to this digestive segment (PREDICTED: mucin-5AC [Meleagris gallopavo], GeneID 723979 and Phospholipase A2 group IB precursor [Gallus gallus], GeneID 416980). Mucin has been associated with intestinal homeostasis as a mucus component that protects the epithelium and aids the passage of feed components all along the digestive tract^28^. Phospholipase A2 is a pancreatic protein involved in lipid metabolism^29^. The presence of this protein was previously reported in the jejunum of broilers fed a corn distillers’ dried grain with soluble, a soybean meal, or a pea diet but not in the jejunum of chickens fed a rapeseed diet, which was further confirmed by the present study. Retention time in the proventricular/gizzard has been reported to vary between 30 minutes to two hours^30^, which is in accordance with our current results where the maximum number of proteins (from both *Brassica napus* and chicken) was identified in group B (1h30 after feed withdrawal, Fig. 4a,b).

Duodenum, jejunum and ileum are all parts of the small intestine. Duodenum is the first segment of this digestive structure and receives secretions from both pancreas and gall bladder, resulting in pH rising (Fig. 1). As compared with other segments, it contains the highest number of proteins (highest protein concentration, Supplementary Figure 2 and highest protein number, Fig. 2) and most of the proteins identified in this segment are duodenum-specific (Fig. 4c). Its protein profile is much more complex as compared with other digestive segments (Fig. 3a). Some proteases are present in this segment as demonstrated by the proteolytic activity (zymography analysis) and mass spectrometry results. However, proteolytic enzymes represent only around 20% of the total proteins (A: 12/60, B: 13/56, C: 9/44, Fig. 5). Most proteins identified in this segment refer to lipid metabolism (Fatty acid-binding protein, intestinal, GeneID 422678; Pancreatic lipase, partial, GeneID 423916) carbohydrate metabolism (Pancreatic alpha-amylase precursor, GeneID 414140; PREDICTED: sucrase-isomaltase, intestinal, GeneID 425007; PREDICTED: maltase-glucoamylase, intestinal, GeneID 425545; Acidic mammalian chitinase precursor, GeneID 395072; Triosephosphate isomerase; Transaldolase, GeneID 423019; Aldehyde dehydrogenase, mitochondrial, GeneID 101802897; Fructose-bisphosphate aldolase B, GeneID 427308; Transketolase, GeneID 102090087), hormone metabolism (Calreticulin, GeneID 100859104; Hematopoietic prostaglandin D synthase, GeneID 395863) and vitamin metabolism (Retinol-binding protein 2, GeneID 424822) (Supplementary dataset 1, sheet 3). Moreover, half of these proteins are associated with tissue homeostasis/response to stress (Supplementary dataset 1, sheet 3). Thereby, the duodenum appears as particularly sensitive/responsive to stress and is probably involved in the overall maintenance of the functionality of the whole small intestine. When considering the passage rate of feed nutrients in this segment, only two *Brassica*-derived proteins could be identified three hours after feed withdrawal (Group C), whereas 17 different proteins were identified at the time of feed withdrawal (Group A), which suggests that most feed proteins have passed through this segment after three hours (Fig. 4b).

Jejunum and ileum share several similarities. Protein concentration (Supplementary Figure 2) and number of proteins identified are in the same range in the two digestive segments (Fig. 4), SDS-PAGE and zymography profiles are very comparable although the overall proteolytic activity is higher in the ileum (Fig. 3b). In both digestive segments, proteases represent around 60–70% of the total number of proteins identified (Fig. 5). The majority of the proteins identified were also detected in the other digestive segments but six were specific to these segments (four in the jejunum and two in the ileum, Fig. 4c). Neutral ceramidase (GeneID 423679) is a protein recovered in the intestine and involved in sphingolipid metabolism. It is not specific to the jejunum segment but a previous study in mice indicates that jejunum is the segment with the highest expression^31^. Several immunoglobulins were found, particularly in jejunum, belatedly in the course of digestion (jejunum or ileum, Group C only), and may result from transudation from serum, or as a result of local production and release. These immunoglobulins are readily resistant to pepsin and trypsin digestion which could explain their presence in the distal part of the digestive tract^32^.

*Brassica*-derived proteins that remained in the digesta in these digestive segments are likely dietary proteins that are poorly digestible and that have not been fully solubilized by digestive enzymes (Table 1, Supplementary dataset 2). Proteins identified at the ileal level even three hours after feed withdrawal are mainly cruciferin and trypsin inhibitors. Napins were detectable in the crop and proventriculus/gizzard segments even three hours after the end of feeding but not in the other digestive segments, which confirm previous observations done in broilers^7^. However, for these seven proteins, the sequence coverage ranges from 5% to 22% (Supplementary Figure 3), which indicates that these proteins might be partially degraded. Consequently, these proteins are not fully indigestible, which may be due to their three-dimensional structure, the presence of disulfide bonds, or their association to with feed components. These results suggest that their digestion might be harder and longer.

Additionally, the presence of anti-nutritional components such as protease inhibitors (Rapeseed trypsin inhibitor 3 (CAJ44307.1), Trypsin inhibitor DE-3-like (XP_013713143.1), Table 1) is assumed to impair the function of digestive enzymes. These components that disturb the proper digestive processes also challenge the gut, thus inducing a higher need in maintaining intestinal homeostasis. Interactions between the feed and the microbiota have not been studied in the current article but it is assumed that the microbial profile of the gut may also deeply affect feed utilization by the animal^33^.

To our knowledge, this work provides the most comprehensive view of the proteins/proteases involved in chicken digestion in each distinct digestive segment. It also highlighted the presence of new rapeseed proteins that resist digestive proteolysis, which may help to identify levers to facilitate digestion of rapeseed-based diets by chickens, including the selection of new rapeseed varieties devoided of such antinutritional factors and/or the development of protease additives. We also believe that such results could potentially be transposed to others monogastric species. Indeed, up to 80% of the endogenous proteins identified in the chicken digestive tract have homologs in both human and pig species (supplementary dataset 1, sheets 2 and 3), while 20% have no homologs identified yet in these two species and do not have any reported activity to date (except four that are related to immune response (immunoglobulins) and two, to regulation of proteolysis (Trypsin, geneID 103533306 and serpin B6, gene ID 420895, supplementary dataset 1, sheets 2 and 3)). These findings suggest that the molecular players recruited for protein digestion, metabolism and homeostasis may be essentially the same between chicken, pig and human species and thereby, that the chicken may constitute a good consensus model to decipher the molecular mechanisms of digestion in monogastric animals.

## Methods

Procedures involving the use of animals were approved by the Centre Val de Loire French ethics committee C2EA -19 (approval number no. 2013-01-4). All experiments were conducted according to the European legislation on the “protection of animals used for experimental and other scientific purposes” set by the European community Council Directive of November 24, 1986 (86/609/ECC).

### Experimental design and sampling

**Figure 6:**
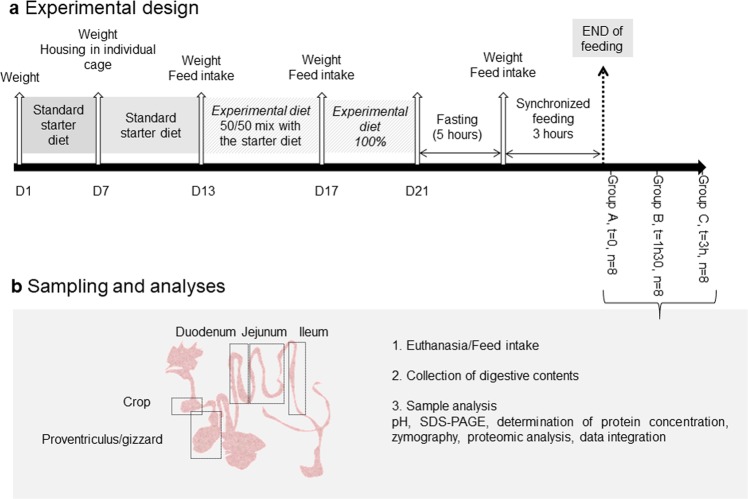
Diagram describing the experimental design and analyses performed on collected samples.

The experimental design is illustrated in Fig. 6. Ross PM3 broiler chicks (n = 24) were purchased from a commercial hatchery (Grelier, Saint-Laurent-de-la-Plaine, France). From days 1 to 7, chicks were housed on floor and were fed a standard starter diet. On day 7, chicks were housed in individual cage. A semi-synthetic experimental diet containing rapeseed meal (56.50%) was formulated. Energy was supplied by a mix of corn starch (22.43%), sucrose (11.05%) and soya seed oil (5.0%). Titanium dioxide (Ti0_2_, 0.50%) was added as an indigestible marker. Between days 13 and 17, the experimental diet was supplied as a 50/50 mix with the starter diet. Between days 17 and 21, birds were fed 100% of the experimental diet. Diet was pelleted at a temperature of 55 °C. Pelleted diet (2.5 mm in diameter) and water were provided ad libitum. Birds were kept under 24 h of light until day 3. Light period was then progressively decreased to reach 18 h of light at day 9. The room temperature was 32 °C on day 1 and was progressively decreased until reaching 24 °C on day 17.

Birds were weighed on days 1, 7, 13, 17 and 21. Feed intake, body weight gain and feed conversion ratio were measured weekly. Based on the body weight on day 17 and the body weight gain between day 13 and day 17, birds were randomly allocated to three homogeneous groups (A, B and C; n = 8) for euthanasia and samples collection. On day 21, all birds were individually weighed after five hour of fasting. After three hours of feeding to ensure that all birds composing each group have a synchronized physiological status, birds were euthanized by a lethal injection of sodium pentobarbital (1 mL per kg of body weight) at the wing vein. The birds were euthanized at i) A: 0 mn, ii) B: 1h30 and iii) C: 3h00 after the end of synchronized feeding. Fasting and synchronized feeding ensured 1) a similar feed intake among birds and 2) lower inter-individual variability due to differences in digestion time. The feed intake over the 3h of feeding was measured. Each digestive segment (n = 24) was collected, emptied from digestive content and weighed (crop = 5.1 ± 0.8 g; proventriculus/gizzard = 21 ± 1.9 g; duodenum = 10.8 ± 2.4 g; jejunum = 19.1 ± 3.61; ileum = 11.1 ± 1.7 g). Contents from the different segments of the digestive tract: crop, proventriculus/gizzard, duodenum, jejunum, and ileum were collected by gentle squeezing. Within each digestive tract segment, the pH of digesta was measured with a SevenGoTM SG2 pH-meter and InLabbs Solids Pro electrode Mettler Toledo, Viroflay, France). Samples were stored at −80 °C until further analyses.

### Apparent ileal digestibility of nitrogen

The nitrogen and TiO_2_ contents were measured in diet and distal ileum digesta^34^.

The apparent ileal digestibility of nitrogen was then calculated as follows:

$${\rm{Apparent}}\,{\rm{Ileal}}\,{\rm{Digestibility}}\,{\rm{of}}\,{\rm{Nitrogen}}( \% )=(1-\frac{Nitrogen\,diet( \% )}{Nitrogen\,digesta( \% )}\ast \frac{Ti{O}_{2}\,digesta( \% )}{Ti{O}_{2}\,diet( \% )})\ast 100$$

### Protein analyses

Digesta samples were prepared as described in a previous publication^6^. For each sample, the weight of the sample portion taken, the buffer (initial weight) and the weight of the remaining pellet obtained after centrifugation and that of the supernatant (final weight) were recorded. The weight of the insoluble part of the sample was expressed as the percentage of the initial weight as follows:

$$Weight\,of\,the\,inso{luble}\,part( \% )=\frac{Final\,weight(g)}{Initial\,weight(g)}\ast 100$$

Protein concentration in the supernatant was determined using the Dc-Biorad Assay (Bio-Rad, Marnes-la-Coquette, France), with bovine serum albumin (Interchim, Montluçon, France) as the standard. SDS-PAGE analyses were performed as previously described^6^. After checking the homogeneity of each sample by individual analyses (Supplementary Figure 4), the supernatants were pooled within digestive tract segment and group of euthanasia (8 birds per condition). The proteolytic activity was assessed by gelatin zymography^35^. After migration onto SDS-PAGE under non reducing and non-heating conditions, the gels were incubated for 1 hour at 41 °C in activation buffer (0.2 M di-sodium hydrogen Phosphate, citric acid, pH 4.2 or 5.3 or 0.5 M Tris-HCl, pH 6.2 or 8.2) was adapted to fit with physiological values measured in each digestive tract segment). After staining with Coomassie blue R250, hydrolytic bands appear as clear bands on a blue background.

### Mass Spectrometry

Samples (diet, n = 1 and digesta, n = 15 corresponding to each A, B, C group in each digestive segment) were loaded on a running gel without fractionation. After inclusion in gel (30 mn, 50 V), proteins concentrated in one single band for each sample were stained with Coomassie blue and the band was further excised and analyzed by nano-liquid chromatography tandem mass spectrometry (nanoLC-MS/MS) as previously described^36^. Gel pieces were washed in water: acetonitrile solution followed by 100% acetonitrile. Briefly, reduction and cysteine alkylation was performed by successive incubation with dithiothreitol and then iodoacetamide both in 50 mM NH4HCO3. Proteolytic digestion was carried out overnight using 25 mM NH4HCO3 with 12.5 ng/μl Trypsin (Sequencing grade, Roche diagnostics, Paris, France). Resulting peptides were extracted by incubation in 5% formic acid, followed by incubation in 100% acetonitrile and 1% formic acid (1:1, 10 min) and a final incubation with acetonitrile (5 min). These two peptide extractions were pooled and dried using a SPD1010 speedvac system (Thermosavant, Thermofisher Scientific, Bremen, Germany) and the peptide mixture was then analyzed by nanoLC-MS/MS. All experiments were performed on a dual linear ion trap Fourier Transform Mass Spectrometer (FT-MS) LTQ Orbitrap Velos (Thermo Fisher Scientific, Bremen, Germany) coupled to an Ultimate® 3000 RSLC Ultra High Pressure Liquid Chromatographer controlled by Chromeleon Software (Thermo Fisher Scientific, Bremen, Germany). Samples were desalted and concentrated using LCPackings trap column (Acclaim PepMap 100 C18, 75 µm inner diameter × 2 cm long, 3 µm particles, 100 Å pores). The peptide separation was conducted using a LCPackings nano-column (Acclaim PepMap C18, 75 µm inner diameter × 50 cm long, 2 µm particles, 100 Å pores) at 300 nL/min. Columns equilibration was performed with 96% solvent A (0.1% formic acid, 97.9% water, 2% acetonitrile (v/v/v)) and 4% solvent B (0.1% formic acid, 15.9% water, 84% acetonitrile (v/v/v)). A gradient of 4–60% solvent B for 90 min and a stage at 99% solvent B for 15 min were applied. Data were acquired in positive mode in data-dependent mode to automatically switch between high resolution full-scan MS spectra (Resolution set at 60 000) and low resolution CID-MS/MS (m/z 300-1800). The 20 most intense peptide ions with charge states ≥2 were sequentially isolated and fragmented in the high pressure linear ion trap by CID fragmentation mode. Dynamic exclusion was activated during 30 seconds with a repeat count of 1. MS/MS ion searches were performed using Mascot search engine version 2.3.2 (Matrix Science, London, UK) via Proteome Discoverer 2.1 software (ThermoFisher Scientific, Bremen, Germany) against NCBIprot_Brassica, NCBIprot_Chordata and NCBIprot_Bacteria databases (July 2017). The search parameters included trypsin as a protease with two allowed missed cleavages and carbamidomethylcysteine, methionine oxidation and acetylation of N-term protein as variable modifications. The tolerance of the ions was set to 5 ppm for precursors and 0.8 Da for fragment ions. Peptides and proteins identified by MASCOT were validated using ≪ Peptide Prophet ≫ and ≪ Protein Prophet ≫ algorithm with Scaffold software (version 4.8.3, Proteome Software, Portland, USA). Protein identifications were accepted if they contained at least two identified exclusive unique peptide. The abundance of identified proteins was estimated by calculating the emPAI (Exponentially Modified Protein Abundance Index) using Scaffold Q+ software (version 4.8.3, Proteome Software, Portland, USA).

### Statistical analyses

Statistical analyses were performed with R software (RStudio Team 2016). The effect of the group of euthanasia and/or the digestive tract segment on the feed intake over the 3 h of synchronized feeding, the digesta pH, the protein concentration in the supernatants and the weight of the insoluble part of samples were analyzed by Generalized Linear Models or the non-parametric test of Kruskal-Wallis depending on data distribution (determined by the Shapiro-Wilk test). Difference was considered significant at *P* < *0.05*. Post-hoc multiple comparisons tests of Tukey or Kruskal-Wallis were used accordingly.

## Supplementary information

Supplementary figures 1-4

Supplementary dataset 1

Supplementary dataset 2
