# Supplementary material for: Digestion dynamics in broilers fed rapeseed meal
Source: Sci Rep. 2019 Feb 28;9:3052. doi: 10.1038/s41598-019-38725-1 (PMC6395701; doi:10.1038/s41598-019-38725-1)
Supplement: Supplementary file 1 — Supplementary figures 1-4 [file 41598_2019_38725_MOESM1_ESM.docx]

**Digestion dynamics in broilers fed rapeseed meal**

E. Recoules, M. Lessire, V. Labas, MJ. Duclos, L. Combes-Soia, L. Lardic, C. Peyronnet, A. Quinsac, A. Narcy and S. Réhault-Godbert

**Supplementary figures 1, 2, 3, 4**

**
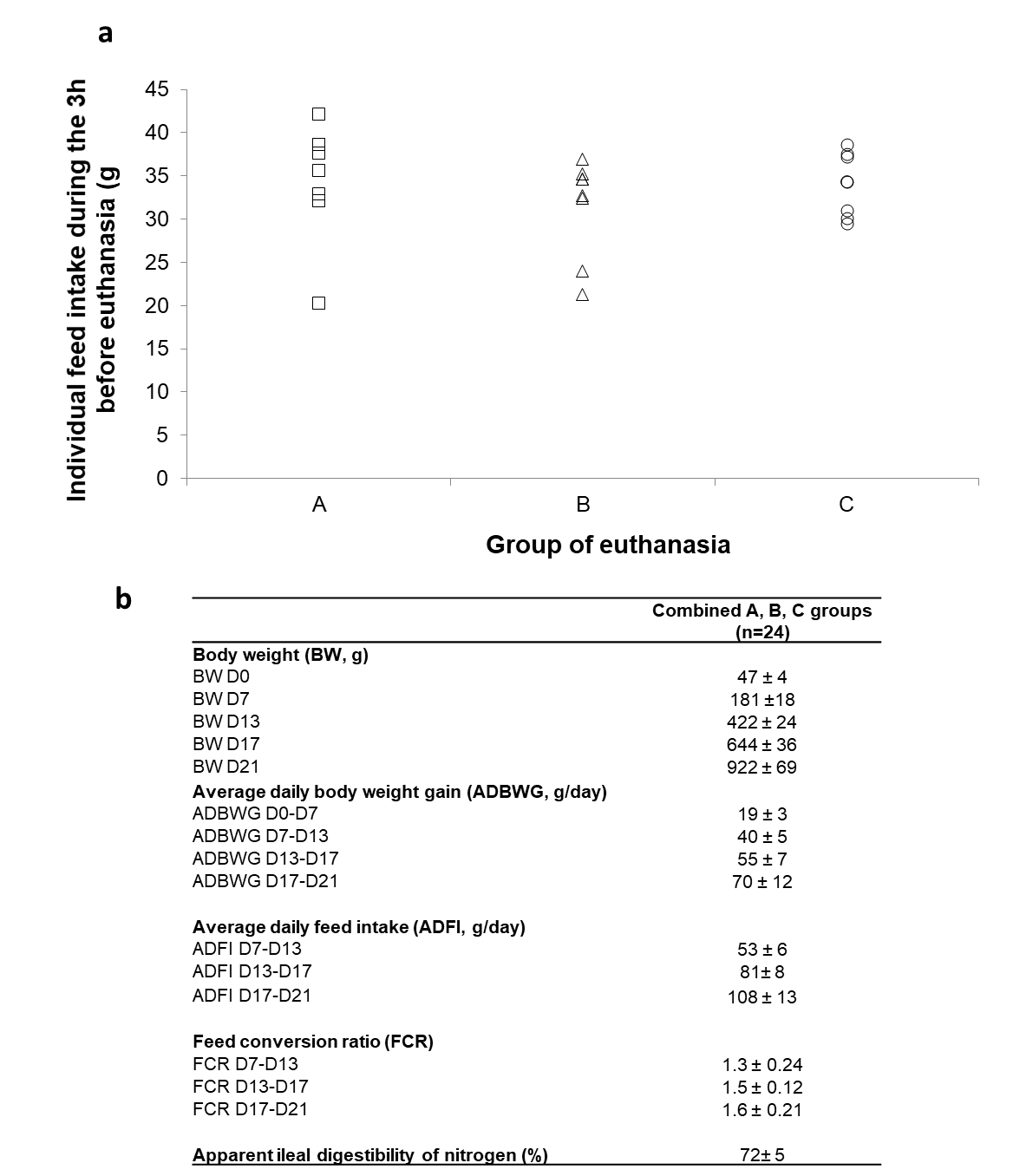
**

**Supplementary figure 1**. **a** Total feed intake during the 3-hour synchronized feeding. A, t=0; B, t=1h30; C, t=3h after the end of the 3-hour synchronized feeding. **b** animals’ performance

**
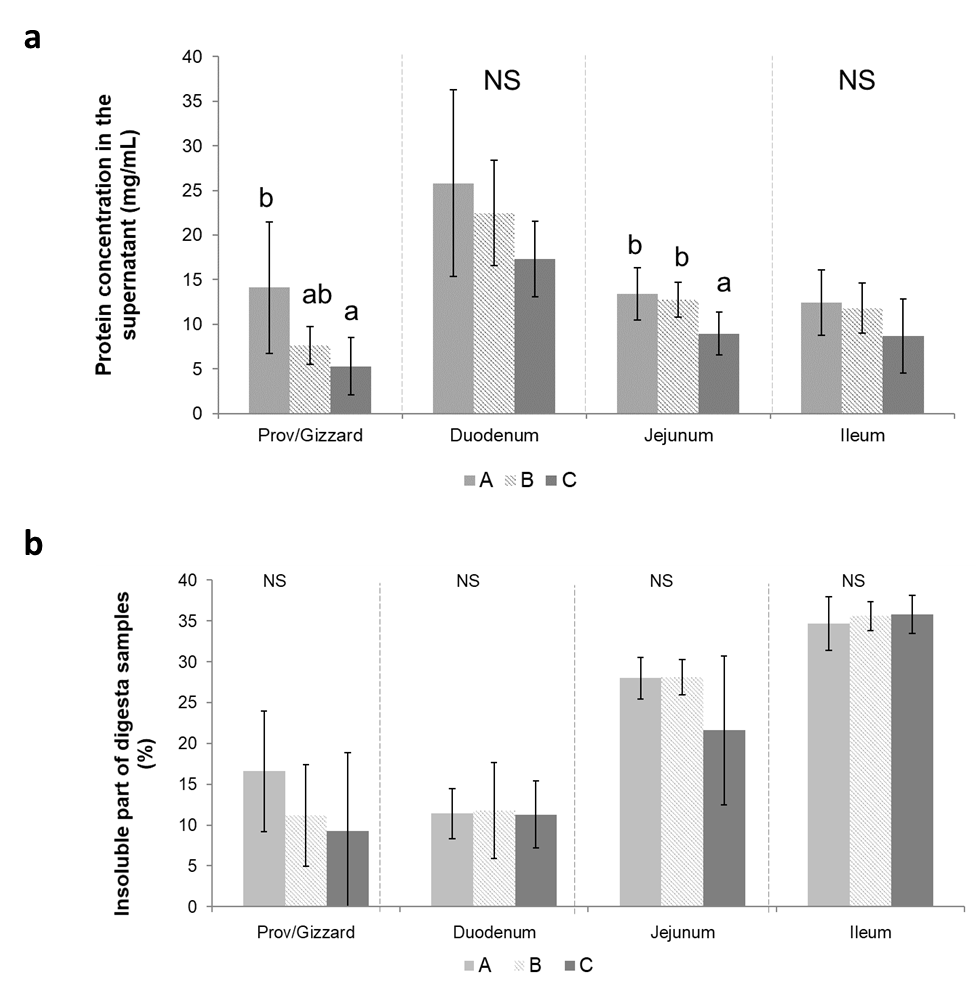
**

**Supplementary figure 2**. Protein concentration of hydrosoluble fractions in each digestive tract (**a**) and relative content of the insoluble content (**b**) in groups A, B and C. Protein concentration (mg/mL) of each insoluble fraction (groups A, B, C) was determined using Dc-Biorad Assay (Bio-Rad, Marnes-la-Coquette, France) as described in Methods. The remaining insoluble fraction obtained after centrifugation and removal of hydrosoluble fraction was expressed as a percentage of the initial weight (group A, B, C)

| **Protein** | **Accession number** | **Molecular weight (kDa)** | **emPAI** | **Number of AA identified** | **Total number of AA** | **Sequence coverage/**  **(%)** | **Peptides Start – Stop indexes** |
| --- | --- | --- | --- | --- | --- | --- | --- |
| BnaA08g13680D | CDY14237.1 | 55 | 2.48 | 109 | 493 | 22 | 78-95  96-108  335-350  356-367  385-402  447-465  469-480 |
| PREDICTED: trypsin inhibitor DE-3-like | XP_013713143.1 | 24 | 1.88 | 42 | 218 | 19 | 64-77  78-94  145-155 |
| GDSL esterase/lipase At1g54020-like precursor | NP_001302825.1 | 42 | 1.27 | 51 | 371 | 14 | 57-66  187-201  202-217  241-250 |
| PREDICTED: putative phosphatidylglycerol/phosphatidylinositol transfer protein DDB_G0282179 | XP_013714672.1 | 16 | 1.26 | 20 | 152 | 13 | 34-53  39-53 |
| BnaA09g04300D | CDY19777.1 | 39 | 1.04 | 39 | 344 | 11 | 157-170  200-214  306-314 |
| Myrosinase-binding protein | AAC08048.1 | 99 | 0.78 | 49 | 956 | 5 | 276-288  364-373  553-564  618-631 |
| BnaA06g36310D/cruciferin precursor | CDY22309.1 | 51 | 0.55 | 55 | 462 | 12 | 77-88  363-385  416-435 |

**Supplementary figure 3:** Sequence coverage of dietary proteins identified in the ileum


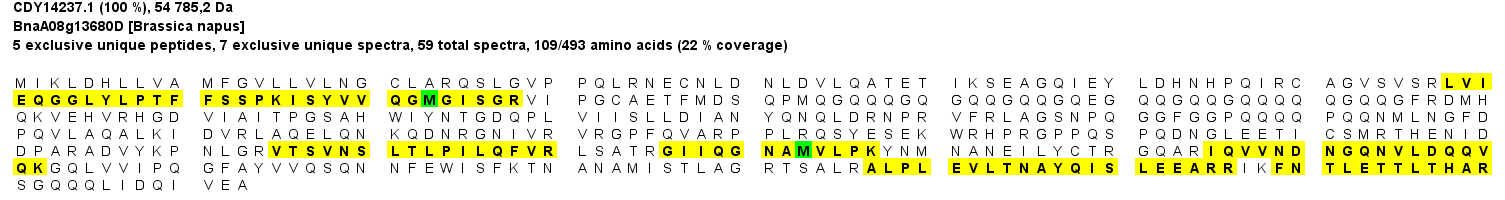


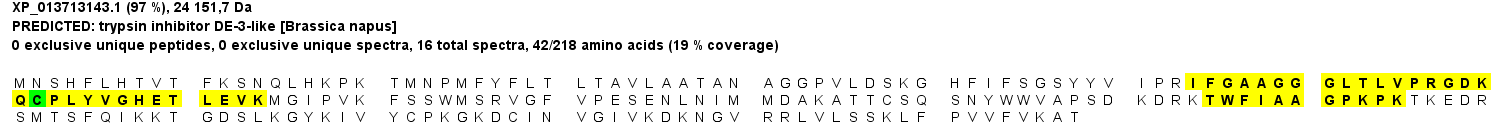


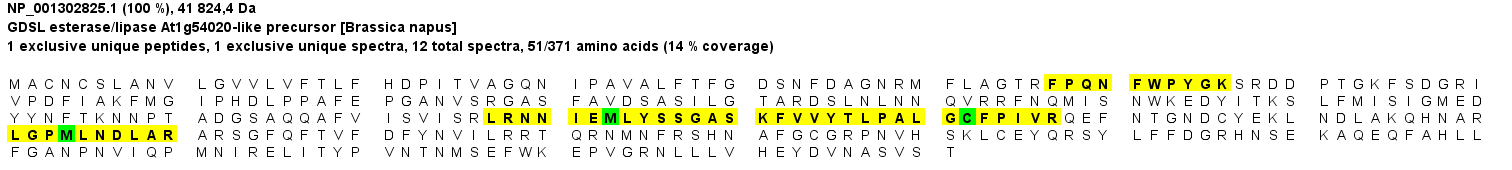


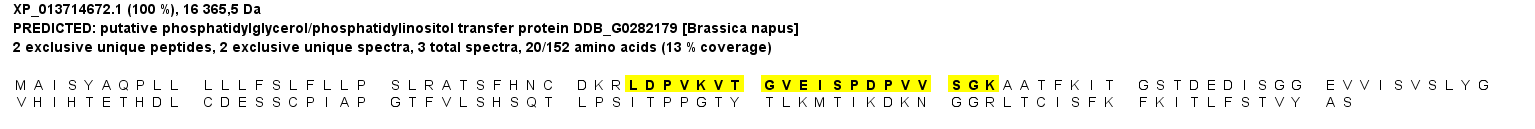


**Supplementary figure 3:** Sequence coverage of dietary proteins identified in the ileum (continued)


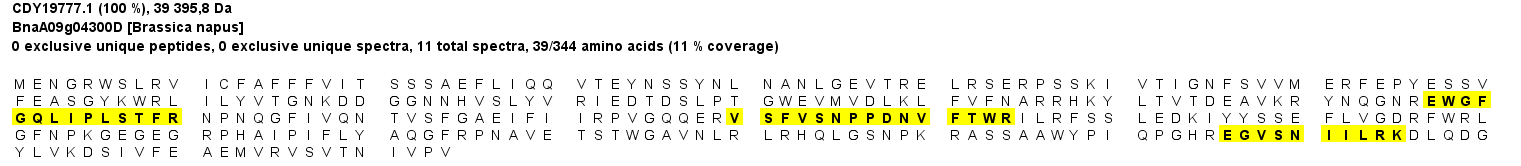


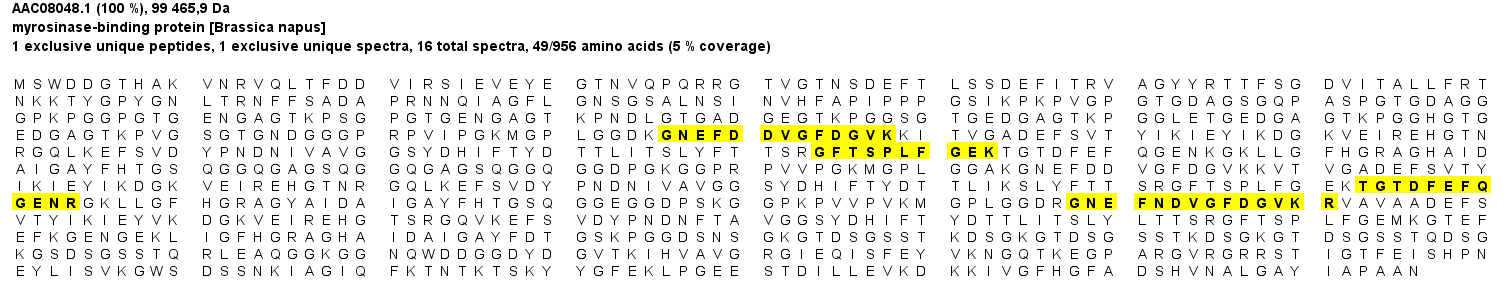


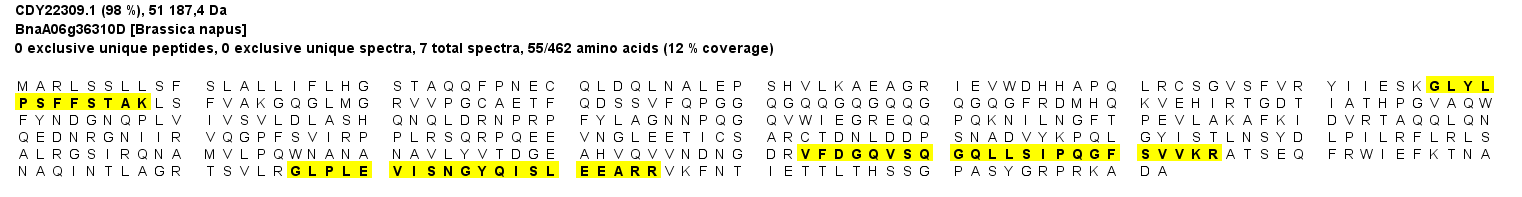


**Supplementary figure 3:** Sequence coverage of dietary proteins identified in the ileum (continued)


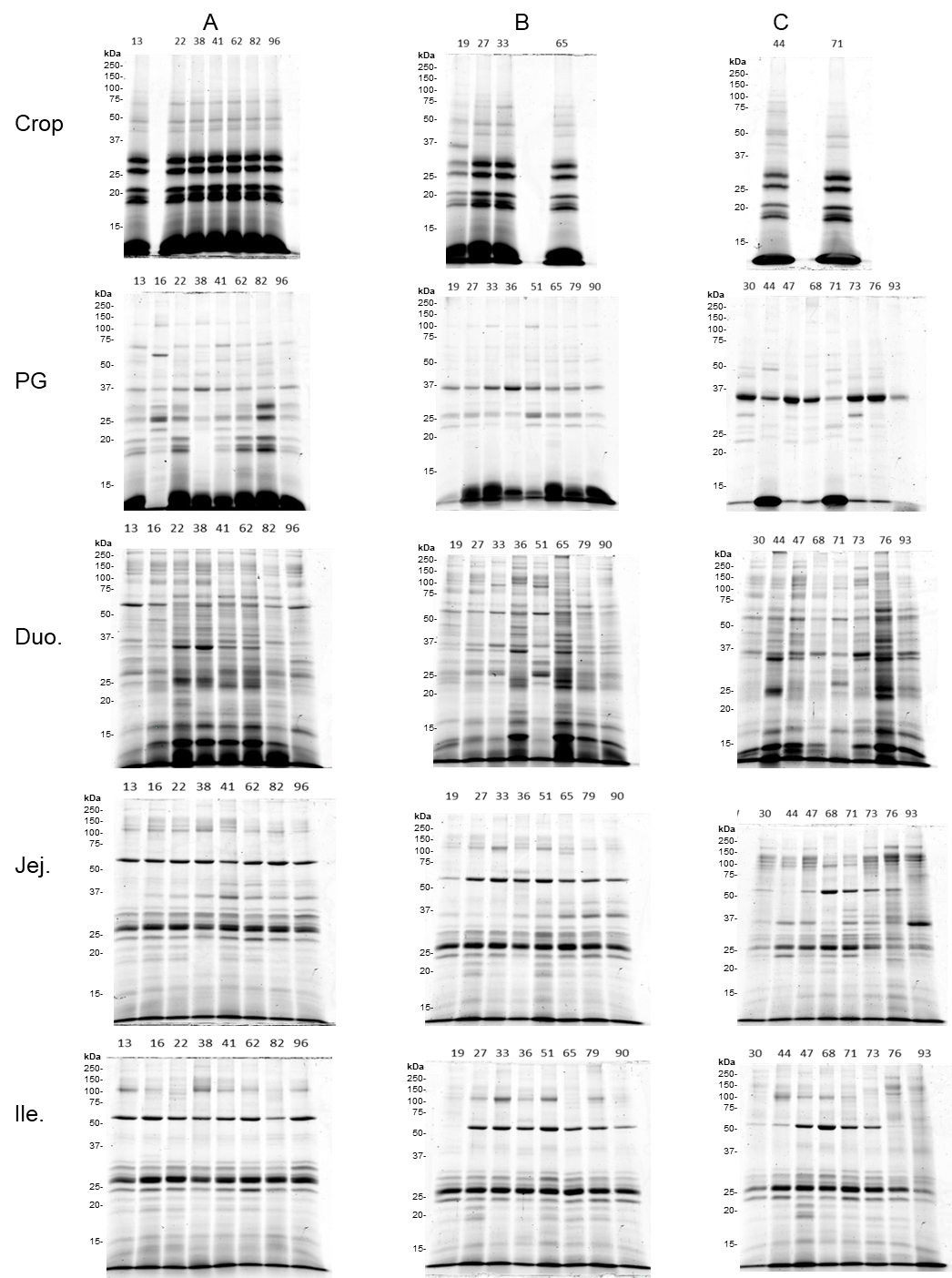


**Supplementary figure 4:** SDS-PAGE of individual samples. Note that some crop samples were missing (crop lacking content)
